# Dose-dependent protective effects of theophylline on testicular endocrine function in a rat model of ischemia-reperfusion injury

**DOI:** 10.3389/fphar.2026.1814726

**Published:** 2026-06-17

**Authors:** Duygu Demiriz Gulmez, Alparslan Apan, Ozgun Cuvas Apan, Murat Usta, Esma Cinar

**Affiliations:** 1 Department of Anesthesiology and Reanimation, University of Health Sciences Başakşehir Çam and Sakura City Hospital, Istanbul, Türkiye; 2 Department of Anesthesiology and Reanimation, Giresun University School of Medicine, Giresun, Türkiye; 3 Department of Biochemistry, Giresun University School of Medicine, Giresun, Türkiye; 4 Department of Pathology, Giresun University School of Medicine, Giresun, Türkiye

**Keywords:** ischemia–reperfusion injury, rat model, testicular torsion, testosterone, theophylline, TNF-α

## Abstract

**Introduction:**

Testicular torsion (TT) induces ischemia–reperfusion (I/R) damage, frequently resulting in inflammatory responses, oxidative stress, and compromised spermatogenesis. This study investigated the potential therapeutic role of theophylline—a methylxanthine with established antioxidant and anti-inflammatory properties—on testicular I/R outcomes in a rat model.

**Methods:**

Thirty-two pubertal Wistar–Albino rats were categorized into four cohorts (n=8 per group): Control, I/R, and I/R treated with either 10 mg/kg or 50 mg/kg theophylline.

**Results:**

While plasma biochemical markers (tumor necrosis factor-alpha (TNF-α), glutathione, myeloperoxidase, ischemia-modified albumin, inhibin B, caspase-3, and testosterone) and histopathological Johnsen tubular biopsy scores remained statistically comparable across groups, significant intergroup variations were observed in testicular tissue TNF-α (p = 0.017), testicular testosterone (p = 0.003), and Cosentino histopathological grades (p < 0.05).Although high-dose theophylline showed a downward trend in tissue TNF-α, this specific pairwise difference did not reach statistical significance.

**Discussion:**

These results indicate that while acute structural protection was limited within a 4-hour reperfusion window, high-dose theophylline demonstrates a promising capacity to preserve testicular testosterone levels and modulate local inflammatory markers. This suggests a dose-dependent supportive effect on Leydig cell function and endocrine recovery following ischemic insult.

## Introduction

As a critical urological emergency, testicular torsion (TT) primarily affects neonates and adolescents, with an estimated incidence of 1 in 4,000 males under 25 (Wei et al., 2020; Yildirim et al., 2018). The condition stems from the rotation of the spermatic cord, which triggers acute ischemia; subsequent detorsion often exacerbates tissue damage through ischemia–reperfusion (I/R) injury (Li et al., 2026). Research indicates that torsion exceeding 4–6 h can lead to permanent cellular degradation and infertility (Embaby et al., 2026). The severity of this injury is heavily influenced by the degree of cord rotation, with 720° torsion causing more profound clinical damage than lesser rotations (Vaos and Zavras, 2017).

Pathophysiologically, I/R injury involves a complex cascade of reactive oxygen species (ROS) production, neutrophil recruitment, and inflammatory cytokine activation (Yildirim et al., 2018). Due to their high polyunsaturated fatty acid content, testicular germ cells are exceptionally vulnerable to such oxidative stress (Wei et al., 2020). Theophylline, a phosphodiesterase inhibitor, has shown promise in modulating adenosine pathways and reducing ischemic damage in various organs (Aslankara et al., 2018; Sato et al., 1998). Various pharmacological agents have been investigated to mitigate testicular I/R injury in experimental models (Acer-Demir et al., 2020; Aragones et al., 2008; Keseroglu et al., 2019). However, its efficacy in the specific context of testicular I/R remains unexplored.

The objective of this research was to determine the efficacy of varying theophylline doses in modulating the biochemical and pathological consequences of testicular reperfusion injury.

## Materials and methods

### Animals and ethical considerations

The experiment involved 32 male Wistar–Albino rats, aged approximately 7–8 weeks at the start of the protocol which corresponds to the late pubertal/young adult stage, each weighing between 250 and 350 g. Subjects were sourced from the “Saki Yenilli Laboratory for Experimental Animal Production and Applications,” a facility with official accreditation. Rats were maintained under controlled laboratory settings, including a 12-h light–dark cycle and *ad libitum* access to standard chow and water. Protocol approval was granted by the Giresun University Animal Ethics Committee (Decision No: 2019/01, dated 18/02/2018), and all procedures adhered to institutional welfare guidelines. The study design followed the ARRIVE (Animal Research: Reporting of *In Vivo* Experiments) framework.

### Experimental design and grouping

Subjects were randomly distributed into four distinct experimental cohorts (n = 8 per group).

- Group 1 (Control): A sham procedure was performed where the right testis was exposed via scrotal incision and immediately replaced without torsion.
- Group 2 (I/R): Testicular torsion was maintained for 2 h, followed by detorsion and a subsequent 4-h reperfusion period.
- Group 3 (I/R + 10 mg/kg Theophylline): Following the 2-h torsion, 10 mg/kg of theophylline was administered intraperitoneally (IP) 60 min prior to the initiation of detorsion.
- Group 4 (I/R + 50 mg/kg Theophylline): The same I/R protocol was applied, with a higher theophylline dose (50 mg/kg) administered IP 1 h before detorsion.

Dosage selection for theophylline was informed by existing literature regarding its pharmacological safety and efficacy in rat models (Sato et al., 1998).

### Surgical procedures

Anesthesia was induced using an IP combination of ketamine (80 mg/kg) and xylazine hydrochloride (10 mg/kg), with supplemental half-doses provided as necessary to maintain deep anesthesia. Following aseptic preparation, the right testis was mobilized through a scrotal approach. In torsion-assigned groups, the right testis was rotated 720° in a clockwise direction and secured to the scrotal wall using silk sutures to prevent spontaneous detorsion. After the 120-min ischemic phase, the testis was returned to its natural anatomical position to allow for 4 h of reperfusion (Figure 1). Humane euthanasia was performed post-reperfusion using manual cervical dislocation under deep anesthesia (confirmed by the absence of reflexes), followed by a right orchiectomy for subsequent analysis. At the time of euthanasia, rats weighed between 250 and 350 g.

**FIGURE 1:**
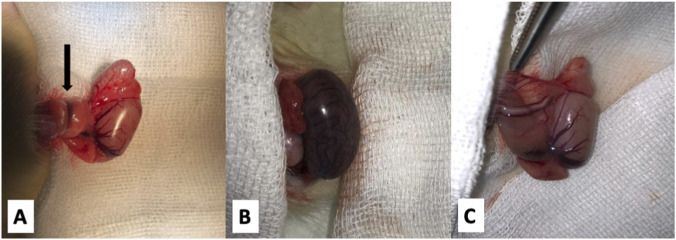
Macroscopic evaluation of the experimental testicular ischemia–reperfusion model **(A)** Intraoperative appearance of the right testis after induction of 720° clockwise spermatic cord torsion. Black arrow shows the torsion point **(B)** Gross appearance of the right testis following 2 h of ischemia, demonstrating marked discoloration and congestion consistent with ischemic changes **(C)** Right testis after detorsion and 4 h of reperfusion, showing partial restoration of color with persistent vascular congestion.

### Biochemical and histopathological assessment

At the conclusion of the 4-h reperfusion period, blood samples (approximately 3–5 mL) were collected via cardiac puncture under deep anesthesia. The samples were immediately transferred into tubes containing heparin as an anticoagulant before being processed by centrifugation at 1,000 × g (4 °C) for 20 min to isolate plasma., which was then stored at −80 °C. Testicular tissues were homogenized in phosphate-buffered saline (100 mg tissue/1 mL PBS), and the resulting supernatants were preserved at −80 °C. Levels of markers including tumor necrosis factor-alpha (TNF-α), glutathione, myeloperoxidase (MPO), ischemia-modified albumin (IMA), inhibin B, caspase-3, and testosterone were quantified using enzyme-linked immunosorbent assay (ELISA) (USCNK, Wuhan USCN Business Co., Ltd., Hubei 430056, PRC; Catalog No: SEA133RA, SEA601RA, CEA825RA, 703002, CEA760RA, SEA626RA, CEA458GE) and electrochemiluminescence immunoassay (ECLIA) cobas® 8,000 modular analyzer series (Roche Diagnostics, United States) kits as per the manufacturer’s protocols. For histopathology, tissues were fixed in 10% formalin, paraffin-embedded, and cut into 5 µm sections for H&E staining. Microscopic analysis was conducted by blinded observers using a consensus-based approach to determine the Cosentino grading system (Cosentino et al., 1986) and the Johnsen tubular biopsy score (JTBS).

### Statistical analysis

Statistical analyses were performed using SPSS version 26.0 (IBM Corp., Armonk, NY, United States). Data distribution was assessed using the Kolmogorov–Smirnov test. Quantitative variables were expressed as mean ± standard deviation or median (range), as appropriate. Comparisons among groups were conducted using the Kruskal–Wallis test, followed by the Mann–Whitney U test for pairwise comparisons. Categorical variables were analyzed using the chi-square test. A p-value <0.05 was considered statistically significant.

## Results

Testicular ischemia was successfully induced in all experimental groups subjected to torsion, with no intraoperative complications observed. Following detorsion and reperfusion, all animals survived until the planned orchiectomy.

No statistically significant differences were observed among Groups 1–4 in plasma levels of TNF-α, glutathione, MPO, inhibin B, or IMA (p > 0.05) (Table 1). Similarly, plasma testosterone levels did not differ significantly among the control and I/R groups, although higher values were observed in the high-dose theophylline group (Table 1).

**TABLE 1:** Biochemical parameters of experimental groups.

Parameters	Group I (Control)	Group II (I/R)	Group III (I/R + theo 10 mg/kg)	Group IV (I/R + theo 50 mg/kg)	p-value
Plasma TNF-α (pg/mL)	25.3 ± 23.4	26.8 ± 22.7	29.4 ± 16.7	19.2 ± 3.1	0.629^K^
Plasma Glutathione (µg/mL)	222.1 ± 87.7	252.3 ± 71.5	214.9 ± 33.1	173.9 ± 71.1	0.384^K^
Plasma Testosterone (ng/mL)	0.29 ± 0.20	0.38 ± 0.09	0.63 ± 0.37	1.33 ± 1.67	0.095^K^
Plasma MPO (ng/mL)	76.4 ± 10.1	75.2 ± 16.3	85.4 ± 33.9	84.6 ± 18.1	0.788^K^
Plasma Inhibin B (pg/mL)	25.5 ± 15.2	31.9 ± 12.9	34.2 ± 21.6	37.1 ± 45.9	0.657^K^
Plasma IMA (ng/mL)	18.4 ± 2.6	19.6 ± 3.4	19.8 ± 0.8	20.0 ± 3.1	0.584^K^
Testicular TNF-α (pg/mg protein)	313.5 ± 124.1	496.2 ± 66.1	515.5 ± 129.1	445.8 ± 115.7	**0.017** ^K^
Testicular Glutathione (µg/mg protein)	3.2 ± 1.3	3.2 ± 0.9	3.7 ± 1.5	4.3 ± 1.1	0.162^K^
Testicular Testosterone (ng/mg protein)	1.7 ± 1.0	3.5 ± 3.5	3.2 ± 1.6	6.9 ± 2.9	**0.003** ^K^
Testicular Caspase-3 (ng/mg protein)	1.4 ± 0.8	1.8 ± 0.4	1.5 ± 0.4	1.5 ± 0.5	0.309^K^

Data are presented as mean ± standard deviation.

^K^ Kruskal–Wallis (Mann-Whitney U test).

I/R: ischemia–reperfusion; Theo: theophylline; MPO: myeloperoxidase; IMA: ischemia-modified albumin; TNF-α: tumor necrosis factor-alpha.

Bold values indicate statistical significance (p < 0.05).

Biochemical evaluation of testicular tissue demonstrated statistically significant intergroup differences in TNF-α levels (p = 0.017). However, no significant variations were detected in tissue glutathione or caspase-3 concentrations (p > 0.05) (Table 1). Notably, high-dose theophylline (Group 4) significantly preserved testicular testosterone levels compared to both untreated I/R group (p = 0.021) and the low-dose treatment group (p = 0.012), as detailed in the pairwise comparison matrix (Table 2). No significant differences in testosterone were found between Groups 1, 2, and 3. Although the high-dose theophylline group showed a tendency toward reduced TNF-α levels compared to the untreated I/R groups, this specific pairwise difference did not reach statistical significance (Table 1).

**TABLE 2:** Pairwise comparison matrix of significant testicular biochemical parameters.

Comparison	Testicular TNF-α (p)	Testicular Testosterone (p)
Group I vs. Group II	**0.006**	0.093
Group I vs. Group III	**0.018**	0.093
Group I vs. Group IV	**0.048**	**0.002**
Group II vs. Group III	0.565	1.000
Group II vs. Group IV	0.180	**0.021**
Group III vs. Group IV	0.277	**0.012**

*p*-values were calculated using the Mann-Whitney U test as a *post hoc* analysis for variables demonstrating significant intergroup variation in the Kruskal–Wallis test. Group I: control; Group II: Ischemia-Reperfusion (I/R); Group III: I/R + Theophylline 10 mg/kg; Group IV: I/R + Theophylline 50 mg/kg. Bold values indicate statistical significance (p < 0.05).

Histopathological evaluation using the Cosentino grading system revealed higher Grade 3 scores in the Group 4 compared to other groups (p < 0.05). However, JTBS values were comparable across all groups (p > 0.05) (Table 3).

**TABLE 3:** Histopathological assessment according to the Cosentino grading system and the Johnsen tubular biopsy score (JTBS).

Grading system	Grades	Group I (Control) N = 8	Group II (I/R) N = 8	Group III (I/R + theo 10 mg/kg) N = 8	Group IV (I/R + theo 50 mg/kg) N = 8	p-value
Cosentino	Grade I	8 (100.0%)	8 (100.0%)	5 (62.5%)	2 (25.0%)	**p < 0.05** ^**X2**^
	Grade II	0 (0.0%)	0 (0.0%)	3 (37.5%)	2 (25.0%)	
	Grade III	0 (0.0%)	0 (0.0%)	0 (0.0%)	4 (50.0%)	
JTBS	Score 10	8 (100.0%)	8 (100.0%)	8 (100.0%)	6 (75.0%)	p > 0.05 ^**X2**^
	Score 9	0 (0.0%)	0 (0.0%)	0 (0.0%)	1 (12.5%)	
	Score 8	0 (0.0%)	0 (0.0%)	0 (0.0%)	1 (12.5%)	

Variables are presented as number (percentage). *p*-values were calculated using Chi-square test (Fischer exact test). Bold values indicate statistical significance (*p* < 0.05).

Representative H&E–stained sections demonstrated edema, congestion and mononuclear inflammation in the interstitial area in the I/R groups (Figure 2). These findings were not significantly attenuated by theophylline treatment within the short reperfusion period.

**FIGURE 2:**
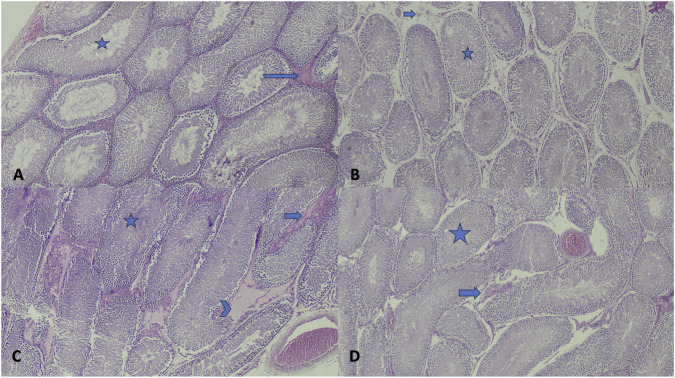
Histological evaluation of the hematoxylin and eosin–stained sections of testicular tissue (original magnification ×40) **(A)** Group I (Control), Grade 1: Normal tubular architecture and normal germinal epithelium. The asterisk (*) indicates a seminiferous tubule, and the arrow points to the interstitial area **(B)** Group II (I/R), Grade 1: Preserved tubular architecture with marked edema in the interstitial area (arrow). Asterisk (*): seminiferous tubule **(C)** Group III (I/R + theophylline 10 mg/kg), Grade 1: Preserved tubular architecture with mild mononuclear inflammation in the interstitial area (arrow). The arrowhead points to serum accumulation, while the asterisk (*) indicates a seminiferous tubule **(D)** Group IV (I/R + theophylline 50 mg/kg), Grade 3: Irregular seminiferous tubule architecture with interstitial edema, congestion, chronic inflammation (arrow), hemorrhage, and pyknotic germinal cell nuclei. Asterisk (*): seminiferous tubule.

## Discussion

TT is a urological emergency that results in acute interruption of blood flow to the testis and may lead to irreversible tissue damage and impaired fertility if not treated promptly (Embaby et al., 2026). Although surgical detorsion restores perfusion, the subsequent I/R process itself may exacerbate tissue injury through oxidative stress and inflammatory mechanisms.

In the present study, the effects of two different doses of theophylline were evaluated in an experimental model of testicular I/R injury. While most biochemical and histopathological parameters did not differ significantly among the groups, administration of high-dose theophylline (50 mg/kg) was associated with a significant increase in testicular testosterone levels compared with untreated and low-dose groups. This finding suggests a possible dose-dependent supportive effect of theophylline on testicular endocrine function.

Previous experimental studies have demonstrated that the extent of testicular injury following torsion depends on both the duration of ischemia and the degree of spermatic cord rotation (Abbaszadeh et al., 2020; Chen et al., 2020). In our model, 720° torsion for 2 h followed by a 4-h reperfusion period was selected to induce measurable ischemic injury without causing extensive necrosis. Consistent with prior reports, overt histological recovery was not expected within this limited reperfusion window (Acer-Demir et al., 2020). In our study, the high-dose theophylline group exhibited the most robust preservation of intratesticular testosterone levels, despite manifesting higher Cosentino Grade 3 scores. We interpret this finding as an early-stage functional-structural dissociation. The effective maintenance of testosterone within the acute 4-h reperfusion window suggests that high-dose theophylline successfully supports Leydig cell viability and endocrine output. This functional resilience likely stems from the ability of theophylline and its derivatives to elevate intracellular cAMP levels, thereby activating survival pathways such as PI3K/Akt and regulating cellular functions in gonadal tissues (Zhang et al., 2024). However, because structural remodeling in ischemia-reperfusion models often lags behind biochemical recovery, this functional protection may not have translated into observable histological improvement at this early time point. Furthermore, the fact that JTBS remained stable and comparable across all torsion groups indicates that the underlying spermatogenic architecture remained intact. This suggests that the elevated Cosentino scores in the theophylline treated group reflect transient interstitial edema or vascular congestion, likely due to the drug’s dose dependent vasodilatory effects, rather than irreversible tissue damage. When integrated with the JTBS and testosterone data, these results demonstrate that high dose theophylline provides significant functional protection that precedes architectural normalization.

Testosterone plays a crucial role in maintaining spermatogenesis and germ cell survival. Reduced testosterone levels have been associated with impaired testicular recovery following ischemic injury. The observed increase in testicular testosterone levels in the high-dose theophylline group may indicate preserved Leydig cell function or enhanced resistance to ischemic damage. Although this hormonal change did not translate into significant histopathological improvement within the short reperfusion period, it may represent an early indicator of functional recovery.

The inflammatory cascade, characterized by cytokine production (specifically TNF-α) and neutrophil recruitment, plays a pivotal role in the pathogenesis of testicular I/R damage. In the present study, while no differences were found in plasma TNF-α levels, significant variations were observed in testicular tissue TNF-α concentrations. This distinction suggests that the inflammatory response during the acute phase of reperfusion remains localized within the tissue rather than manifesting as a systemic elevation.

The observed downward trend in tissue TNF-α levels within the high-dose theophylline group—despite not achieving significance in pairwise comparisons—points toward the potential anti-inflammatory capacity of theophylline. These results align with previous reports demonstrating theophylline’s ability to modulate inflammatory pathways in other organ systems (Sato et al., 1998; Şahin et al., 2011). However, the lack of a definitive statistically significant reduction suggests that the 4-h reperfusion window may be too brief to fully capture the anti-inflammatory impact on testicular architecture. This highlights the importance of the dose-time relationship when evaluating pharmacological interventions for TT.

Several pharmacological agents with antioxidant and anti-inflammatory properties have been investigated in experimental testicular I/R injury models, with varying degrees of success (Aragones et al., 2008; Abbaszadeh et al., 2020; Chen et al., 2020; Syu et al., 2025; Oliveira et al., 2024; Minas et al., 2023). Similar to previous studies evaluating agents such as N-acetylcysteine, ibuprofen, and hydrogen sulfide donors, the lack of statistically significant histological improvement in the present study highlights the challenges of demonstrating tissue-level protection during the acute reperfusion phase (Keseroglu et al., 2019).

### Limitations

Several constraints of this research should be acknowledged. Primarily, the modest sample size might have restricted the statistical power necessary to identify minor variations between the experimental cohorts. Second, the reperfusion period was short and may not have been sufficient to reveal delayed biochemical or histopathological changes. Third, only two doses of theophylline were evaluated, and functional fertility outcomes such as sperm count or motility were not assessed. Finally, as an experimental animal study, the findings may not be directly translatable to clinical practice.

## Conclusion

In conclusion, our study demonstrates that while significant histopathological changes were not fully reversed within a short 4-h reperfusion window, high-dose theophylline (50 mg/kg) exerts a potent protective effect on testicular endocrine function by significantly preserving testosterone levels. The observed significant variation in local TNF-α levels, coupled with the hormonal recovery in the high-dose group, suggests that theophylline modulates the early inflammatory and functional landscape of I/R injury in a dose-dependent manner. These findings highlight theophylline as a promising pharmacological candidate for mitigating Leydig cell dysfunction following TT. Further long-term studies focusing on spermatogenesis and fertility outcomes are warranted to translate these experimental insights into clinical strategies for testicular salvage.

## Data Availability

The original contributions presented in the study are included in the article/supplementary material, further inquiries can be directed to the corresponding author.
